# What is the relationship between sclerostin and cardiovascular events in hemodialysis patients?

**DOI:** 10.1080/0886022X.2020.1845733

**Published:** 2020-11-16

**Authors:** Yu Zhao, Wenyun Wang, Zhilong Dong

**Affiliations:** a Department of Medicine, Northwest University for Nationalities, Lanzhou, PR China; b Department of Pediatric Surgery, Second Hospital of Lanzhou University, Lanzhou, PR China; c Department of Urology, Second Hospital of Lanzhou University, Lanzhou, PR China

Dear Sir,

We have read with great interest the paper entitled ‘Association of sclerostin with cardiovascular events and mortality in dialysis patients’ by Zou et al. [1]. Sclerostin is a 22 kDa anti-anabolic protein that is produced by osteocytes and suppresses osteoblast activity *via* inhibition of canonical Wnt/β-catenin signaling. The article included 165 dialysis patients (84 hemodialysis [HD] and 81 peritoneal dialysis[PD]) from China with a median serum sclerostin level of 250.9 pg/ml by using R&D ELISA kits, and showed that both overall and cardiovascular events (CVEs)-free survival rates were significantly lower in the high serum sclerostin group(>250.9 pg/ml) compared to the low serum sclerostin group (<250.9 pg/ml) in patients with PD (*p* < 0.05) by the Kaplan–Meier analysis. A low serum sclerostin was associated with better overall survival and lower prevalence of CVEs in patients with PD. However, in HD patients, the Kaplan–Meier analysis showed that only CVE-free survival rates notably declined in the high serum sclerostin group compared to the low serum sclerostin group(*p* = 0.029). Cox proportional hazard regression models showed that serum sclerostin levels were not associated with all-cause mortality (RR = 0.967, *p* = 0.926) or CVEs (RR = 1.164, *p* = 0.509) in HD patients. I pay special attention to the results of this research because it caused me some confusion. Therefore, we cite several studies to compare and contrast with the finding of Yun et al.

Firstly, Gonçalves et al. [2] evaluated the relationship between serum sclerostin and all-cause mortality in HD patients. Ninety-one patients from Brazil aged 42.4 ± 18.8 years with a mean serum sclerostin of 0.88 ng/ml by using TecoMedical (TE) ELISA kits were included. Competing risk regression showed that sclerostin levels were positively associated with increased mortality over the follow-up period (HR 2.18, 95% CI 1.41–3.38, *p* < 0.001). When sclerostin levels were categorized as high (>0.88 ng/ml) and low (<0.88 ng/ml) while keeping all other variables, high sclerostin (>0.88 ng/ml) were associated with a HR 2.88 (95% CI 1.35–6.15, *p* < 0.05). After the additive model exploration, there was an association between all-cause mortality and high sclerostin (HR 2.2, 95%CI 1.35–3.56, *p* = 0.001). Serum sclerostin is an independent predictor of mortality in HD patients.

Secondly, a cross-sectional study conducted by Jean et al. [3] 207 HD patients from France with a mean serum sclerostin level of 1.9 ± 0.7 ng/ml by using TE ELISA kits who were divided into three groups (tertile 1 0.6-1.53 ng/ml; tertile 2 1.58-2.2 ng/ml; tertile 3 2.2-4.6 ng/ml), and showed that patients of the 3rd tertile (2.2-4.6 ng/ml) displayed a lower mortality rate compared to tertile 1 using multivariable-adjusted Cox model (HR 0.5, 95%CI 0.25–0.93, *p* = 0.03). Higher serum sclerostin levels are associated with a better survival rate. Drechsler et al. [4] conducted a prospective cohort study of 673 dialysis patients (614 HD patients and 59 PD patients) from the Netherlands with a mean serum sclerostin 1.24 ± 0.57 ng/ml by using TE ELISA kits showed that patients in the highest sclerostin tertile (>1.37 ng/ml) had a significantly lower risk of cardiovascular death (HR 0.29, 95% CI 0.22–0.68, *p* < 0.05) and for all-cause mortality (HR 0.39, 95% CI 0.22–0.68, *p* < 0.05) compared with patients of the lowest tertile (≤0.95 ng/ml).

Finally, Kalousová et al. [5] conducted a prospective observational cohort study consisted of 106 HD patients from 2 dialysis centers in University hospitals in the Czech Republic and 25 healthy subjects for 5-year follow-up. Sclerostin was measured in the serum using Biomedica ELISA kit. Serum sclerostin concentrations were higher in HD patients compared to the controls (89.2 ± 40.3 pmol/l vs 32.8 ± 13.0, *p* < 0.001). A higher cardiovascular risk was connected to sclerostin concentrations above the median (>84 pmol/l), HR 2.577 (95% CI 1.002–10.207, *p* = 0.04). High sclerostin levels were demonstrated as a risk factor for cardiovascular mortality.

In conclusion, I think the conflicting results may be secondary to the use of different sclerostin assays, different populations, and variable dialysis treatment modalities. The consistency of testing methods should be standardized. Further prospective studies of larger dialysis populations with a longer follow-up time are needed to confirm this relationship.

Yu Zhao *Department of Medicine, Northwest University for Nationalities, Lanzhou, PR China* Wenyun Wang *Department of Pediatric Surgery, Second Hospital of Lanzhou University, Lanzhou, PR China* Zhilong Dong *Department of Urology, Second Hospital of Lanzhou University, Lanzhou, PR China* 562691313@qq.com
